# Sickle cell mice exhibit mechanical allodynia and enhanced responsiveness in light touch cutaneous mechanoreceptors

**DOI:** 10.1186/1744-8069-8-62

**Published:** 2012-09-10

**Authors:** Sheldon R Garrison, Audra A Kramer, Nashaat Z Gerges, Cheryl A Hillery, Cheryl L Stucky

**Affiliations:** 1 Department of Cell Biology, Neurobiology and Anatomy, Medical College of Wisconsin, 8701 Watertown Plank Road, Milwaukee, WI, USA; 2 Department of Pediatrics, Division of Hematology/Oncology, Children’s Research Institute, Medical College of Wisconsin, Milwaukee, WI, USA; 3 Blood Research Institute, BloodCenter of Wisconsin, Milwaukee, WI, USA

**Keywords:** Primary afferents, Allodynia, Pain, Nociception, Anxiety, Open field test

## Abstract

**Background:**

Sickle cell disease (SCD) is associated with both acute vaso-occlusive painful events as well as chronic pain syndromes, including heightened sensitivity to touch. We have previously shown that mice with severe SCD (HbSS mice; express 100% human sickle hemoglobin in red blood cells; RBCs) have sensitized nociceptors, which contribute to increased mechanical sensitivity. Yet, the hypersensitivity in these neural populations alone may not fully explain the mechanical allodynia phenotype in mouse and humans.

**Findings:**

Using the Light Touch Behavioral Assay, we found HbSS mice exhibited increased responses to repeated application of both innocuous punctate and dynamic force compared to control HbAA mice (100% normal human hemoglobin). HbSS mice exhibited a 2-fold increase in percent response to a 0.7mN von Frey monofilament when compared to control HbAA mice. Moreover, HbSS mice exhibited a 1.7-fold increase in percent response to the dynamic light touch “puffed” cotton swab stimulus. We further investigated the mechanisms that drive this behavioral phenotype by focusing on the cutaneous sensory neurons that primarily transduce innocuous, light touch. Low threshold cutaneous afferents from HbSS mice exhibited sensitization to mechanical stimuli that manifested as an increase in the number of evoked action potentials to suprathreshold force. Rapidly adapting (RA) Aβ and Aδ D-hair fibers showed the greatest sensitization, each with a 75% increase in suprathreshold firing compared to controls. Slowly adapting (SA) Aβ afferents had a 25% increase in suprathreshold firing compared to HbAA controls.

**Conclusions:**

These novel findings demonstrate mice with severe SCD exhibit mechanical allodynia to both punctate and dynamic light touch and suggest that this behavioral phenotype may be mediated in part by the sensitization of light touch cutaneous afferent fibers to suprathreshold force. These findings indicate that Aβ fibers can be sensitized to mechanical force and should potentially be examined for sensitization in other tissue injury and disease models.

## Findings

Sickle cell disease (SCD) is due to a point mutation in the beta chain of hemoglobin that causes polymerization of deoxyhemoglobin that distorts the shape of erythrocytes, contributing to vascular obstruction and ischemia of tissues and organs [1]. A hallmark feature of the disease is severe pain that arises during acute sickling events, as well as the recently recognized and less well understood chronic pain syndromes that develop in many of these individuals. Patients report heightened sensitivity to touch and spontaneous pain, suggesting complex physiological underpinnings that may include both inflammatory and neuropathic etiologies [2]. The array of pain descriptors and triggers for acute and chronic pain suggest contribution from both central *and* peripheral mechanisms, involving changes in neural signaling, gene expression and plasticity [3]. Primary afferent sensitization, in particular, is a correlate of psychophysical measurements of hyperalgesia, characterized in part by increased responses to suprathreshold peripheral stimuli.

Recently, we reported that mice with severe sickle cell disease, which express only human sickle hemoglobin in circulating erythrocytes (HbSS mice) [4], exhibit marked chronic mechanical hypersensitivity. Histological analysis of sickle mouse skin has revealed increased sensory innervation, elevated calcitonin gene-related peptide and substance P protein levels and diminished skin thickness [3], all of which may contribute to the well-documented hyperalgesia exhibited in these animals. Primary afferent recordings from both Aδ-mechanoreceptor (AM) (high-threshold ≥ 4mN) and unmyelinated C fiber nociceptors showed enhanced mechanically-evoked action potential firing to suprathreshold force in HbSS mice compared to control HbAA mice that express 100% normal human hemoglobin [5]. Yet, it is likely that these neural populations alone do not explain the complex mechanical allodynia phenotype reported in humans [6], particularly because differences in mechanical firing rates between HbSS and controls were observed only at high mechanical forces in nociceptors [5]. It is possible that sensitization of traditionally non-nociceptive Aβ or rapidly-adapting Aδ (D-hair) afferents may be involved, in addition to sensitization of CNS pathways. Because sickle mice were very sensitive to low intensity von Frey thresholds, we asked whether low threshold mechanoreceptors are sensitized.

### Sickle mice exhibit mechanical allodynia

**Figure 1:**
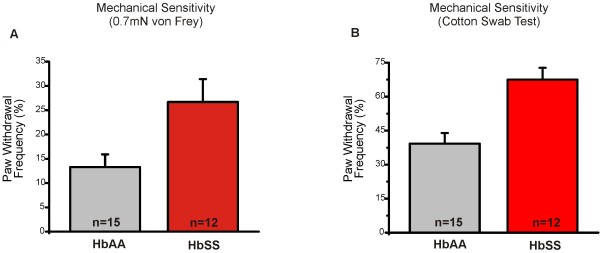
**HbSS mice exhibit increased sensitivity to light-touch mechanical stimuli.** Using the Light Touch Behavioral Assay, mechanical stimuli were applied to the glabrous skin of the hindpaws. The responses of both left and right hindpaws were counted and average to calculate the percent response. (**A**) HbSS mice exhibited a 2-fold increase in paw withdrawal frequency to repeated punctate application of a 0.7mN von Frey monofilament to the plantar hindpaw. HbSS mice responded 26.7 ± 4.7% (n = 12) compared to HbAA controls that responded 13.3 ± 2.6% (n = 15) of the time (**P <0.01). (**B**) Response to light dynamic touch using a repeated <1-sec stroke of a puffed cotton swab resulted in an 1.7-fold increase in response with HbSS mice responding 67.5 ± 5.2% of the time compared to 39.3 ± 4.7% HbAA controls (*P <0.05). Genotypes were compared using student’s t-tests. Error bars indicate S.E.M.

In humans, mechanical allodynia is likely a component of the complex pain associated with SCD [6]. It is not known whether sickle mice exhibit a similar behavioral phenotype. Using our recently developed Light Touch Behavioral Assay [7], we were able to measure allodynia-like behavioral responses to both punctate and dynamic light touch stimuli in sickle mice. We found that HbSS mice exhibited a 2-fold increase in paw withdrawal frequency to repeated application of a 0.7mN von Frey monofilament to the plantar hindpaw (Figure 1A, p < 0.05). The second aspect of the assay recapitulated a dynamic mechanical stimulus, such as light stroke or wind, which is a correlate to increased pain in human SCD patients [6]. Here, we gently stroked a “puffed” cotton swab across the plantar hindpaw skin, and recorded paw withdrawal frequency. HbSS mice exhibited an increased response (1.7-fold) to this dynamic touch (Figure 1B, p < 0.001). Thus, by using two measurements, we were able to demonstrate a mechanical phenotype in sickle mice akin to mechanical allodynia in patients.

### Sickle mice exhibit increased mechanical responsiveness in light-touch primary afferents

While we previously found that nociceptors (myelinated and unmyelinated) in HbSS mice are sensitized to presumably noxious mechanical forces [5], it is possible that light-touch mechanoreceptors are also sensitized and may contribute to mechanical allodynia in SCD. Therefore, we investigated the contribution of the cutaneous Aβ and D-hair afferents, which predominantly transmit non-nociceptive tactile sensation from the periphery. We quantified mechanically-evoked action potentials using the *ex-vivo* saphenous skin-nerve preparation which innervates the hairy skin of the dorsal hindpaw, by recording from single cutaneous fibers and characterized the afferents by their conduction velocity and von Frey thresholds. We then applied increasing sustained force (5-200mN, 10 sec each) to each receptive field to measure firing to suprathreshold stimuli.

**Figure 2:**
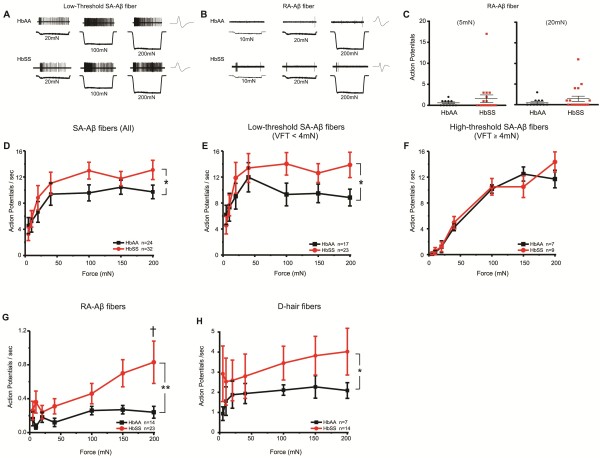
**Mechanically-evoked action potential firing increases in HbSS mouse A-fibers.** Using the skin-nerve preparation, all recordings were performed in the saphenous nerve and hairy skin of the dorsal hindpaw. Mechanical forces ranging 5-200mN (10 sec) were applied to the most sensitive part of each fiber’s receptive field using a 0.8 mm probe. (**A**) Examples of responses of low-threshold slowly adapting Aβ (SA-Aβ) fibers from HbAA and HbSS mice to sustained mechanical force at 20, 100, and 200mN. (**B**) Examples of responses of rapidly adapting Aβ (RA-Aβ) fibers from HbAA and HbSS mice to sustained mechanical force at 10, 20, and 200mN. (**C**) Mechanically-evoked action potentials in at the onset of 5mN and 20mN force in RA-Aβ fibers. Increased firing in a small percentage (20-25%) of HbSS RA-Aβ fibers suggests a possible subpopulation of sensitized fibers. (**D**) Overall, all SA-Aβ fibers firing on average 25% more action potentials to mechanical forces (*P <0.05). (**E**) The differences observed in total SA-Aβ fibers were specific to lower-threshold SA-Aβ fibers, or those with von Frey thresholds < 4mN. On average, the lower-threshold SA-Aβ fibers fired 25% more action potentials in response to mechanical forces in HbSS mice when compared to HbAA controls (*P <0.05). (**F**) The higher-threshold SA-Aβ fibers, which had a von Frey threshold ≥ 4mN, exhibited similar firing properties between HbSS and HbAA mice (*P* >0.05). (**G**) RA-Aβ fibers in HbSS mice fired 75% more action potentials overall (**P <0.01), specifically when compared at 200mN (†). (**H**) Rapidly adapting Aδ (D-hair) fibers responded with markedly more action potentials (75%) when averaged across all force intensities (*P <0.05) in HbSS mice.

The mechanical thresholds for initial action potential responses did not differ for any fiber type in HbSS compared to HbAA control mice (Table 1). In contrast, several fiber types from HbSS mice exhibited sensitization in the form of amplified action potential firing to suprathreshold stimuli. The greatest increase occurred in the rapidly adapting fiber subtypes that likely innervate hair follicles. Rapidly adapting Aβ fibers exhibited an average 75% increase in action potential firing across all force intensities (Figure 2G, p < 0.05). At 200mN, RA-Aβ fibers from HbSS mice fired 3-fold more action potentials than HbAA controls. Additionally, there appears to be a small subpopulation of RA-Aβ fibers in HbSS mice that exhibit increased action potential firing at the onset of mechanical force, although we were unable to identify this small subgroup based on von Frey thresholds or conduction velocity for further electrophysiological testing (Figure 2C). Similarly, the rapidly adapting Aδ D-hair fibers also exhibited a 75% increase in overall firing across all forces (Figure 2H, p < 0.05). On the other hand, the slowly adapting Aβ fibers, many of which innervate Merkel cells, showed a 25% increase in suprathreshold firing (Figure 2D, p < 0.05). We further subtyped the SA-Aβ afferents into lower-threshold (VFT <4mN) and higher-threshold (VFT ≥4mN), because these fiber types exhibit different firing patterns to sustained force [8] and because a small portion of slowly adapting Aβ fibers may be nociceptors [9]. The enhanced mechanical firing in HbSS SA-Aβ afferents was restricted to the lower-threshold SA-Aβ afferents (Figure 2E, p < 0.05). The higher-threshold afferents accounted for a small portion (25%) of total SA-Aβ fibers and exhibited no significant change (Figure 2F p > 0.05). The conduction velocities of all Aβ fiber types did not differ between HbSS and HbAA controls. However, there was a slight, but significant decrease in conduction velocity in D-hair fibers in HbSS mice (Table 1). Taken together, these data suggest that enhanced firing in light touch cutaneous afferents may contribute to the mechanical allodynia-type behavior in mice with sickle cell disease.

**Table 1:** Summary of fiber properties in HbAA and HbSS mice

**Fiber Type**	**Genotype**	**n**	**Median von Frey threshold (mN)**	**Lower Quartile**	**Upper Quartile**	**Mean Conduction Velocity (m/s)**	**±SEM**
**RA-Aβ**	HbAA	17	1.627	0.663	1.627	12.85	0.36
	HbSS	23	1.627	0.270	1.627	14.10	0.60
**SA-Aβ**	HbAA	24	0.663	0.368	4.000	14.49	0.90
	HbSS	32	1.627	0.663	4.000	13.95	0.65
**SA-Aβ Low Threshold**	HbAA	17	0.663	0.270	0.663	15.01	1.19
	HbSS	22	0.663	0.270	1.627	14.69	0.84
**SA-Aβ High Threshold**	HbAA	7	4.000	4.000	4.000	13.24	1.08
	HbSS	9	4.000	4.000	5.410	12.40	0.69
**D-hair**	HbAA	7	0.225	0.225	0.663	6.84	0.29
	HbSS	14	0.270	0.259	0.663	5.04	0.53

### Overall locomotor activity does not correlate with mechanical allodynia in HbSS mice

**Figure 3:**
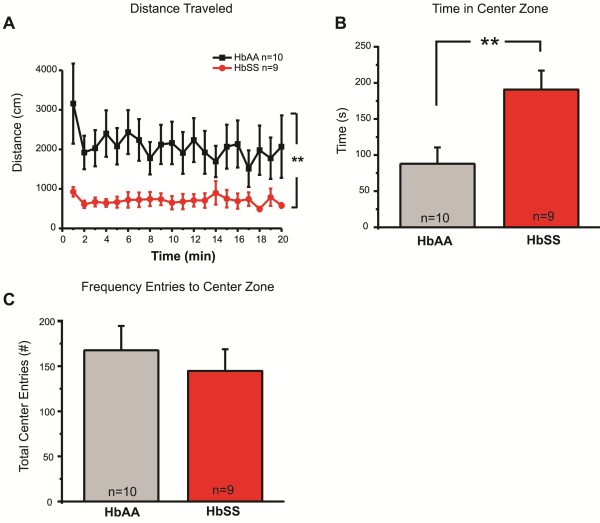
**Decreased locomotor activity in HbSS mice in the open field test.** The open field test was used to measure locomotor activity and anxiety. Animal movement over a 20-minute period was recorded. (**A**) HbSS traveled 706 ± 37 cm (n = 9) compared to HbAA controls that traveled 2081 ± 123 cm (n = 10), a 2.9-fold decrease (**P <0.01). (**B**) Time spent in the center zone averaged 191 ± 26 sec compared to HbAA controls that traveled 88 ± 23 sec. (**C**) Both genotypes exhibited a similar frequency of entries into the center zone. Genotypes were compared using a two-way ANOVA with Bonferroni’s post-hoc analysis (**A**) and student’s t-tests (**B**, **C**). Error bars indicate S.E.M.

To differentiate between an increased reflex response to light mechanical force, and an overall increase in locomotor activity or anxiety levels, we used an open field behavioral assay. HbSS mice exhibited decreased locomotor activity, traveling 65% less than HbAA controls (Figure 3A). We also quantified the amount of time spent in the center zone to measure anxiety-like behavior (Figure 3B). Neither HbAA nor HbSS mice avoided the center zone, indicating that anxiety-like behavior does not contribute to the mechanical hypersensitivity phenotype reported here and elsewhere [3,5]. Additionally, no differences in exploratory behavior (Figure 3C) or immobile time (data not shown) were observed between genotypes. In sum, these data offer compelling evidence that the mechanical allodynia in HbSS mice is independent of other locomotor changes in sickle mice. Although speculative, this may indicate that the HbSS mice experience ongoing pain and move more slowly as a result, similar to the kinesiophobia reported in SCD human patients with heightened pain [10].

This study broadens our understanding of the changes in the somatosensory system in sickle mice that contribute to their behavioral mechanical hypersensitivity. Here we show that several subtypes of low threshold mechanoreceptors that detect innocuous tactile information are sensitized to force in the form of enhanced suprathreshold firing. We and others [3,5] have recently shown that sickle mice exhibit a heightened behavioral sensitivity to traditional von Frey filament threshold measurements, which may be in the noxious range. We also showed that Aδ- and C fiber-type nociceptors are sensitized to mechanical force in the form of enhanced suprathreshold firing [5]. However, sensitized nociceptors, along with central sensitization of CNS pathways, may not fully account for the mechanical allodynia that is prevalent in humans because patients report enhanced sensitivity to wind currents and very light skin touch [6,11]. Therefore, we further investigated the behavioral mechanical phenotype by using a punctate and dynamic light touch assay. Our data show that sickle mice are hypersensitive to very low threshold tactile stimuli. This behavioral phenotype is consistent with SCD-mediated allodynia in human patients and offers a new avenue to identify the cellular and molecular mechanisms that underlie it.

Somatosensory encoding of diverse tactile information from the physical environment is driven by input from a diverse array of mechanoreceptor neurons that are each tuned to detect specific qualities of the stimulus. Rapidly adapting Aβ fibers innervate guard hairs in hairy skin to detect dynamic stimuli such as wind currents, whereas Meissner’s corpuscles are found in the ridges of the glabrous skin where they detect low frequency vibration and microgeometric surface features such as corners and edges. The Aδ D-hair fibers innervate down or vellus hair follicles that are responsible for transmitting very light dynamic stimuli, including soft brush, stroke or light wind currents. Our finding that both types of myelinated hair follicle afferents (RA-Aβ and D-hair) from sickle mice were sensitized to force is interesting in light of the finding that sickle patients report enhanced sensitivity to wind currents, and increased hospitalizations of sickle patients are associated with elevated environmental wind speeds [6,11]. It is possible that these subpopulations of hair follicle afferents are key detectors of environmental wind currents. Slowly adapting Aβ fibers innervate Merkel cells located at the epidermal-dermal border in both hairy and glabrous skin. Merkel cell afferents detect two-point discrimination, textures and patterns of object surfaces. The sensitization of all of these light touch afferent subtypes may contribute enhanced drive to the CNS that facilitates the mechanical hypersensitivity behavioral phenotype in sickle mice.

Importantly, sensitization in the spinal cord and higher brain centers also likely contributes to the behavioral allodynia in sickle mice and the tactile hypersensitivity in patients. Indeed, mechanical allodynia has been long attributed to sensitization of central mechanisms within the spinal cord and higher brain centers. Ongoing activity in nociceptive afferents after nerve injury has been shown to induce sensitization of second order neurons [12] and supraspinal structures [13], independent of putative increased Aβ branching in the spinal cord [14]. The potential sensitization of tactile afferents, including Aβ afferents, after nerve injury or inflammation has largely been dismissed. However, recent evidence has shown that peripheral neuropathy increases both the sensitivity and prolongs the action potential discharge in myelinated Aβ neurons [15,16]. Our findings are similar as they show that in the sickle cell model of chronic mechanical hypersensitivity, Aβ and Aδ tactile afferents exhibit increased action potential firing rates in response to intense mechanical force, regardless of the specific mechanisms that induce this enhanced firing. Importantly, these data highlight the rationale to investigate functional and expression changes in mechanoreceptor molecules expressed in low threshold afferent neurons during any injury or diseases that are associated with persistent or chronic mechanical pain in patients or pain-behavior in animal models.

Enhanced function of the Transient Receptor Potential Vanilloid 1 (TRPV1) channel underlies part of the behavioral hypersensitivity and mediates most of the C fiber nociceptor sensitization to intense force in sickle mice [5]. However, since TRPV1 is not expressed in most non-nociceptive afferents, it is likely that other molecular mechanisms mediate sensitization of light touch myelinated afferents in sickle cell disease. One possibility is that other TRP channels members, such as TRPC1 (Transient Receptor Potential Cannonical 1), may contribute. TRPC1 is functionally expressed in these afferents [17], is important to light touch [7], and has modified channel partner proteins following inflammation [18]. Alternatively, the acid-sensing ion channels (ASIC) have been implicated in mechanical sensitization [19], are expressed in Aβ and Aδ fibers and may be sensitized by the inflammation associated with SCD. Interestingly, the recently described novel family of mechanically sensitive, pore-forming channels, Piezo 1 and 2 [20,21] are plausible mechanotransduction candidates for contributing to the tactile allodynia observed in SCD. Future studies will be essential to determine the molecular mechanism(s) underlying sickle cell tactile allodynia and may open avenues to developing improved therapeutic strategies.

## Materials and methods

### Animals

Berkeley sickle mice (HbSS mice) express 100% human sickle hemoglobin in circulating erythrocytes and mimic many features of the pathobiology of severe SCD in man [4]. Control HbAA mice erythrocytes express only normal human hemoglobin on the same Berkeley background that is null for mouse hemoglobins (*Hba0//Hba0 Hbb0//Hbb0*) [4]. All mice were housed on the same rack and handled prior to and during all behavioral and electrophysiological studies in an identical manner by the same personnel. Adult male mice ages were similar between genotypes, averaging 7.9 ± 0.27 months for behavioral assays and 5.4 ± 0.20 months for skin-nerve recordings (overall range 3–10 months). For electrophysiological recordings, mice were anesthetized by isoflurane and killed by cervical dislocation. All experimental protocols were approved by the Institutional Animal Care and Use Committee of the Medical College of Wisconsin.

### Behavior

Sensitivity to light mechanical force was assessed on the glabrous skin using the Light Touch Behavioral Assay. The assay was performed using dual measures of light touch as previously described [7]. Briefly, for punctate force, the low intensity 0.7mN von Frey filament was applied 10 times to each plantar surface of the hindpaw, alternating between paws. The second assay used a “puffed out” cotton swab to apply a <1-second stroke along the plantar paw surface 5 times. We recorded the number of paw withdrawals in response to both stimulus types [7]. General locomotor and exploratory abnormalities were assessed using the open field test. Mice were handled on three consecutive days prior to testing. On the testing day, mice were placed in the center of a circular chamber (44 cm diameter) and movement was recorded by video camera over a 20 minute time period. Total distance traveled, time spent in center of the arena, and the frequency of entries to center of the arena was analyzed using the EthoVision XL software (Noldus Information Technology, Wageningen, Netherlands). Experimenters were blinded to mouse genotype throughout the collection and analyses of the behavioral data, and to the extent possible during electrophysiological experiments, as enlarged organs in the HbSS mice were sometimes visible during dissection of skin-nerve preparations.

### Teased fiber skin-nerve recordings

The *ex-vivo* saphenous skin-nerve preparation was used to determine mechanical response properties of cutaneous primary afferent fibers in HbSS and HbAA mice following established protocols [8]. Briefly, the saphenous nerve and innervated hairy skin from the dorsal hindpaw was dissected, placed corium side up into the recording chamber and superfused with oxygenated synthetic interstitial fluid at 32 ± 0.5°C [21]. The saphenous nerve was desheathed and fascicles teased apart until functionally single fibers could be distinguished. Mechanically-insensitive units were not included in this study. Fibers were characterized by mechanical threshold using calibrated von Frey filaments (range 0.044-147.0mN) and conduction velocity. Conduction velocity was measured by inserting a Teflon-coated steel needle into the most mechanically-sensitive area of the receptive field and applying square-wave pulses (500 μs), and the action potential latency and the distance between electrodes were quantified. We classified units as Aβ for conduction velocities over 10 m/s, and Aδ for velocities between 1.2-10 m/s. SA fibers responded throughout a sustained mechanical force and adapted slowly to the force, whereas RA fibers responded primarily at the on and offset of force.

Following electrical and mechanical characterization, fibers were recorded for 2-minutes to determine non-stimulus evoked (spontaneous) activity. Next, a feedback-controlled, computer-driven custom mechanical force stimulator was used to apply sustained increasing forces (5, 10, 20, 40, 100, 150 and 200mN) for 10-seconds with 1-minute between force applications to the most mechanically-sensitive area of the receptive field. Action potentials were recorded and analyzed using the LabChart 6data acquisition software (ADInstruments, Colorado Springs, CO).

### Data analysis

Single fiber data was compared between HbSS mice and HbAA controls. For each fiber type, for two groups, mechanical threshold was compared using Mann–Whitney U test for non-parametric data, conduction velocity was compared using student’s t-test. The number of mechanically-evoked action potentials across the force range and distance traveled during the open field test were compared using a two-way ANOVA with Bonferroni post-hoc analysis using the Prism 5 software (GraphPad, La Jolla, CA). For behavioral tests, percent mechanical response, and the open field test time and frequency of entry into the center zone was compared using student’s t-tests for parametric data.

## Competing interests

The authors declare that they have no competing interests.

## Authors’ contributions

All authors read and approved the final manuscript. SG and AK conducted the experiments and analyzed data. SG, CH and CS conceived the study and wrote the manuscript. SG, AK, NG, CH and CS designed experiments and edited the manuscript.
